# Neue Erkenntnisse aus dem RABBIT-Register zum Krebsrisiko von Patient:innen mit rheumatoider Arthritis bei Therapie mit Januskinaseinhibitoren

**DOI:** 10.1007/s00393-025-01740-x

**Published:** 2025-10-20

**Authors:** Martin Schäfer, Anja Strangfeld

**Affiliations:** 1 Programmbereich Epidemiologie und Versorgungsforschung, Deutsches Rheuma-Forschungszentrum Berlin, Charitéplatz 1, 10117 Berlin, Deutschland; 2 Medizinische Klinik mit Schwerpunkt Rheumatologie und Immunologie, Charité-Universitätsmedizin Berlin, Charitéplatz 1, 10117 Berlin, Deutschland

**Keywords:** Malignitätsrisiko, ORAL Surveillance Studie, DMARD-Therapie, Real-World-Evidenz, Prospektive Langzeit-Beobachtungsstudie, Malignancy risk, ORAL Surveillance study, DMARD treatment, Real-world evidence, Prospective longitudinal observational study

## Abstract

Januskinaseinhibitoren (JAKis) sind sehr wirksame Medikamente zur Behandlung entzündlich rheumatischer Erkrankungen. In der randomisierten Studie ORAL Surveillance wurde gezeigt, dass es bei Patient:innen mit rheumatoider Arthritis (RA) im Alter ab 50 Jahren mit mindestens einem weiteren kardiovaskulären Risikofaktor zu einem erhöhten Auftreten bösartiger Erkrankungen bei dem JAKi Tofacitinib im Vergleich zu Tumornekrosefaktorinhibitoren kommt. Dieses Ergebnis wurde in anderen Studien seither jedoch nicht bestätigt. In einer Auswertung des deutschen RABBIT-Registers wurde nun ein insgesamt leicht erhöhtes Krebsrisiko bei jemals mit JAKi behandelten gegenüber jemals mit biologischen (b) krankheitsmodifizierenden Antirheumatika (DMARDs) behandelten RA-Patient:innen beobachtet. Während oder nach 2285 JAKi- und 4259 bDMARD-Behandlungsepisoden traten 88 bzw. 135 bösartige Erkrankungen auf, pro 1000 Patient:innenjahre waren das 11,6 (95 %-Konfidenzintervall (KI): 9,3–14,3) Ereignisse bei JAKis und 8,9 (95%-KI: 7,4–10,5) Ereignisse bei bDMARDs. Das adjustierte Hazard Ratio betrug 1,40 (95%-KI: 1,09–1,80) und war damit jener aus der ORAL Surveillance-Studie vergleichbar. Besonders von der beobachteten Risikoerhöhung betroffen waren Patient:innen ab 60 Jahren, mit einer hohen Krankheitsaktivität oder ≥ 3 früheren Behandlungen mit konventionellen synthetischen DMARDs. Weitere überdurchschnittlich betroffene Gruppen waren Raucher:innen, Männer sowie Patient:innen mit über 10 Jahren RA-Krankheitsdauer. Behandelnde sollten mit Risikopatient:innen sorgfältig gemeinsam abwägen, ob die beobachtete Risikoerhöhung die potenziellen Risiken des Absetzens oder des Vorenthaltens einer hochwirksamen Therapie mit einem JAKi überwiegt – denn auch eine unzureichende Krankheitskontrolle führt zu einem erhöhten Krebsrisiko. Die Ergebnisse aus RABBIT sollten Ärzt:innen im Zweifel nicht davon abhalten, Patient:innen, die eine JAKi-Therapie benötigen, mit dieser zu behandeln. Ein Ausweg könnte vielmehr sein, bei JAKi-Therapie von Risikopatient:innen auf eine engmaschige Krebsvorsorge zu achten.

## Abstract (en)

Janus kinase inhibitors (JAKis) are highly effective drugs for treating inflammatory rheumatic diseases. The randomized ORAL surveillance trial showed that patients with rheumatoid arthritis (RA) aged 50 years and older with at least 1 more cardiovascular risk factor had an increased risk of malignant diseases with the JAKi tofacitinib compared to tumor necrosis factor inhibitors; however, this result has not been replicated in subsequent studies. An analysis of the German RABBIT register now revealed an overall slightly increased cancer risk in RA patients ever treated with JAKi compared to those ever treated with biological (b) disease-modifying antirheumatic drugs (DMARDs). During or after 2285 JAKi treatment episodes and 4259 bDMARD treatment episodes, 88 and 135 malignant diseases occurred, respectively, resulting in a rate of 11.6 (95% confidence interval, CI: 9.3–14.3) events for JAKis and 8.9 (95% CI: 7.4–10.5) events for bDMARDs per 1000 patient years. The adjusted hazard ratio was 1.40 (95% CI: 1.09–1.80), which is comparable to the results from the ORAL surveillance study. Particularly affected by the observed risk increase were patients aged 60 years and older, those with high disease activity and those who had already received ≥ 3 treatments with conventional synthetic DMARDs. Other above-average affected groups included smokers, male patients and patients with an RA duration of > 10 years. When treating patients with an increased risk, clinicians should carefully consider together with them whether this observed risk outweighs the potential risks of discontinuing or withholding a highly effective treatment such as JAKis, bearing in mind that inadequate disease control is also associated with an increased risk of cancer. When in doubt, the results from RABBIT should not discourage physicians from prescribing a JAKi treatment to patients who need it. It could be a way forward to ensure that a close cancer screening is carried out for risk patients treated with JAKis.

## JAKi und die ORAL Surveillance-Studie

Januskinaseinhibitoren (JAKi) sind in Deutschland seit 2017 als sehr wirksame Arzneimittel zur Behandlung verschiedener entzündlich rheumatischer Erkrankungen verfügbar. Bei erwachsenen Patient:innen mit rheumatoider Arthritis (RA) werden sie insbesondere dann eingesetzt, wenn es zuvor bei einem oder mehreren krankheitsmodifizierenden Antirheumatika (DMARDs) zu einem Wirkversagen oder zu Nebenwirkungen kam. In der randomisierten Phase-4-Studie ORAL Surveillance [10] wurde bei Patient:innen mit rheumatoider Arthritis (RA) im Alter ab 50 Jahren mit mindestens einem weiteren kardiovaskulären Risikofaktor ein erhöhtes Auftreten bösartiger Erkrankungen bei dem Januskinaseinhibitor (JAKi) Tofacitinib im Vergleich zu Tumornekrosefaktorinhibitoren (TNFis) festgestellt. Das bereinigte Hazard Ratio (HR) betrug 1,47 (95 %-Konfidenzintervall (KI): 1,00–2,18). Ausgehend von den Ergebnissen der ORAL Surveillance-Studie und vorläufigen Ergebnissen einer Beobachtungsstudie mit Baricitinib, gab die Europäische Arzneimittel-Agentur im Januar 2023 eine Warnung heraus hinsichtlich eines erhöhten Risikos für verschiedene Ereignisse bei Therapie mit JAKis im Vergleich zu TNFis. Die Warnung betraf Patient:innen mit chronischen inflammatorischen Erkrankungen, die bestimmte Charakteristika aufweisen. Diese sind ein Alter ab 65 Jahren, das Vorhandensein von Risikofaktoren für kardiovaskuläre oder maligne Erkrankungen, Rauchkonsum oder eine lange Rauchanamnese [1].

Das in dieser Warnung postulierte erhöhte kardiovaskuläre Risiko bei Therapie mit JAKis wurde aber in keiner der danach durchgeführten Studien bestätigt [4]. Bezüglich des Risikos für Malignome waren die Ergebnisse bislang nicht eindeutig, anhand von Beobachtungsdaten der Routineversorgung konnte ein für JAKi-Therapie erhöhtes Risiko soweit nicht bestätigt werden [2, 3, 5, 6, 9].

## Ergebnisse aus RABBIT konsistent mit klinischer Studie

In einer Auswertung des deutschen RABBIT-Registers [7] wurde nun, ähnlich wie in der ORAL Surveillance-Studie, ein leicht erhöhtes Risiko für ein vermehrtes Auftreten bösartiger Erkrankungen bei jemals mit JAKis behandelten RA-Patient:innen gegenüber jemals mit biologischen (b) DMARDs behandelten RA-Patient:innen beobachtet. Nichtmelanotischer Hautkrebs wurde dabei nicht berücksichtigt. Für die Analyse wurden Behandlungsepisoden analysiert, die zwischen Januar 2017 und Dezember 2020 begonnen und maximal bis Juni 2024 im RABBIT-Register nachverfolgt worden waren. Während oder nach 2285 JAKi- und 4259 bDMARD-Behandlungsepisoden traten 88 bzw. 135 bösartige Erkrankungen auf, pro 1000 Patient:innenjahre waren das 11,6 (95%-KI: 9,3–14,3) Ereignisse bei JAKis und 8,9 (95%-KI: 7,4–10,5) Ereignisse bei bDMARDs. Das um andere Einflussfaktoren wie Alter, Geschlecht, Rauchen, Krankheitsaktivität und vorherige DMARD-Therapien bereinigte HR betrug 1,40 (95%-KI: 1,09–1,80) und war damit jenem aus der ORAL Surveillance-Studie vergleichbar. Es wurde mithilfe von Cox-Regressionsmodellen geschätzt, die mittels inverser Wahrscheinlichkeitsgewichtung adjustiert wurden.

## Geringfügig erhöhtes Krebsrisiko gegenüber Biologika

Bei den JAKi-Behandlungen dominierten in der RABBIT-Analyse die Substanzen Baricitinib und Tofacitinib, während die meisten bDMARD-Behandlungen mittels TNFis erfolgten. Die sog. „number needed to harm“ betrug 368, d. h. um eine zusätzliche Krebserkrankung zu beobachten, müsste man statistisch gesehen 368 Patient:innen für jeweils ein Jahr auf einen JAK-Inhibitor statt auf ein Biologikum einstellen. Dies ist deutlich höher als die sog. „number needed to treat“, die gemäß anderer Studien etwa bei 10 liegt [8]. Dabei handelt es sich um die Anzahl der nicht vollständig auf ein Biologikum ansprechenden Patient:innen (z. B. gemäß der ACR 20 %- und ACR 50 %-Kriterien), die statistisch gesehen mit einem JAK-Inhibitor behandelt werden müssten, damit ein:e zusätzliche:r Patient:in doch noch anspricht. Während in ORAL Surveillance bei einer JAKi-Therapie im Vergleich zu bDMARDs v. a. vermehrt Lungenkarzinome beobachtet wurden, traten in RABBIT eher Fälle von Prostatakrebs sowie gynäkologische Karzinome wie Gebärmutterkörper- und Gebärmutterhalskrebs, Eierstockkrebs und Vulvakarzinome gehäuft auf. Die Fallzahlen für die einzelnen Krebstypen waren jedoch überwiegend klein, sodass hier keine weitergehende Interpretation möglich ist.

## Risiko steigt mit Alter, Krankheitsaktivität und Anzahl der Vortherapien

**Abb. 1:**
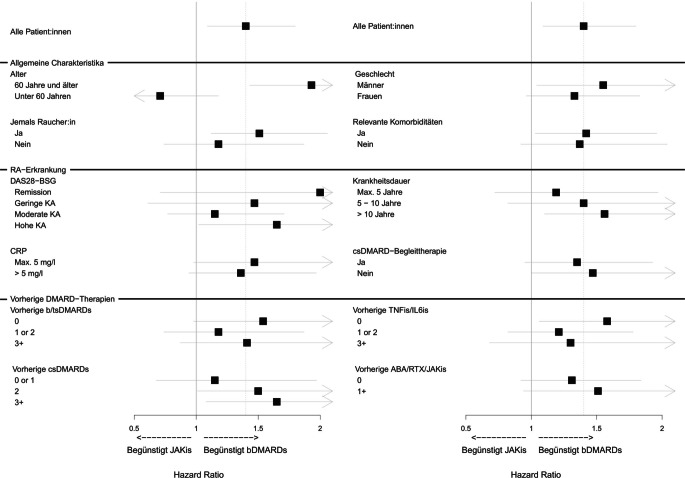
Stratifizierte adjustierte Hazard Ratios für das Auftreten eines Malignoms bei JAKi vs. bDMARD-Therapie [7]. *ABA* abatacept; *bDMARDs* biologische krankheitsmodifizierende Antirheumatika; *CRP* C-reaktives Protein; *csDMARDs* konventionelle synthetische krankheitsmodifizierende Antirheumatika; *KA* Krankheitsaktivität; *DAS28-BSG* Krankheitsaktivitätsscore basierend auf der Blutsenkungsgeschwindigkeit und 28 Gelenken; *IL6is* Interleukin-6-Inhibitoren; *JAKis* Januskinaseinhibitoren; *RA* rheumatoide Arthritis; *RTX* rituximab; *TNFis* Tumornekrosefaktorinhibitoren; *tsDMARDs* zielgerichtete synthetische krankheitsmodifizierende Antirheumatika. Remission ist definiert als DAS28-BSG < 2,6, geringe Krankheitsaktivität als 2,6 ≤ DAS28-BSG ≤ 3,2, moderate Krankheitsaktivität als 3,2 < DAS28-BSG ≤ 5,1 und hohe Krankheitsaktivität als DAS28-BSG > 5,1.

Neben der im Durchschnitt geringen Risikoerhöhung bei JAKis im Vergleich zu bDMARDs wurde für einige Patient:innengruppen eine ausgeprägtere Risikoerhöhung festgestellt. Darunter waren ältere Patient:innen mit Behandlungsbeginn im Alter ab 60 Jahren sowie Patient:innen, die bereits 3 und mehr Behandlungen mit konventionellen synthetischen DMARDs bekommen hatten oder die unter einer besonders hohen Krankheitsaktivität litten (d. h. bei denen der Krankheitsaktivitätsscore basierend auf der Blutsenkungsgeschwindigkeit und 28 Gelenken, DAS28-BSG, mehr als 5,1 Punkte betrug). Weitere überdurchschnittlich von einer Risikoerhöhung betroffene Gruppen waren Raucher:innen, Männer, Patient:innen mit über 10 Jahren RA-Krankheitsdauer sowie Patient:innen, die noch keine TNFis oder Interleukin-6-Inhibitoren erhalten hatten (Abb. 1). Eine besonders ausgeprägte Risikoerhöhung ergab sich für Patient:innen, bei denen ein Alter ab 60 Jahren mit weiteren dieser Faktoren zusammenfiel. Zudem wurde für Patient:innen in DAS28-Remission überraschenderweise eine numerisch deutliche Risikoerhöhung beobachtet, die jedoch nicht statistisch signifikant war. Diese Patient:innen – eine relativ kleine Gruppe – wiesen eine höhere mediane Krankheitsdauer auf als Patient:innen mit moderater oder hoher Krankheitsaktivität (10,7 Jahre vs. 8,8 Jahre) [7]. Es erscheint daher naheliegend, dass nicht Remission selbst einen Risikofaktor darstellt, sondern dass die DAS28-Werte zu Therapiebeginn eine frühere lang anhaltende, potenziell risikobehaftete Krankheitsaktivität nicht abbilden.

## Risiko zeigt sich erst bei längerer Nachbeobachtung

**Abb. 2:**
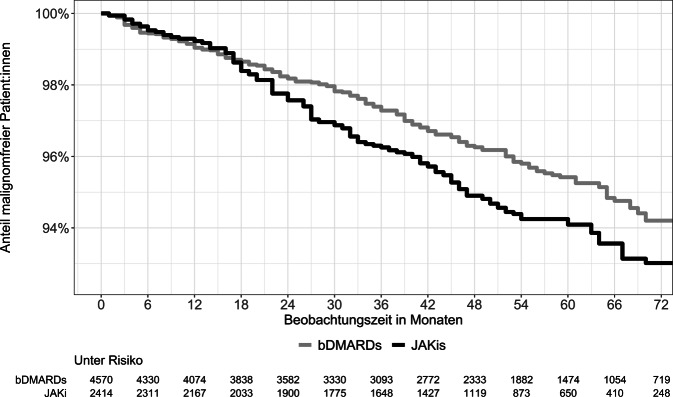
Kaplan-Meier-Kurve: Malignomfreies Überleben bei JAKis und bDMARDs [7]. *bDMARDs* biologische krankheitsmodifizierende Antirheumatika; *JAKis* Januskinaseinhibitoren

Eine Erhöhung des Malignitätsrisikos bei einer JAKi- im Vergleich zu einer bDMARD-Therapie konnte nur beobachtet werden, wenn es eine Nachbeobachtungszeit von mehr als 16 Monaten ab Therapiebeginn gab (Abb. 2). Dies erklärt vermutlich zum Teil, warum andere Studien das Ergebnis aus ORAL Surveillance bisher nicht bestätigen konnten, denn die RABBIT-Analyse ist die erste, die mit etwa 4 Jahren eine vergleichbar lange mediane Nachbeobachtungszeit wie die ORAL Surveillance-Studie aufweist. Andere Studien hatten deutlich kürzere Nachbeobachtungszeiten [2, 3, 6, 9].

## Regelmäßige Krebsvorsorge ist mögliche Alternative zum Absetzen

Die Ergebnisse der RABBIT-Studie können Ärzt:innen dabei helfen, das individuelle Risiko ihrer Patient:innen einzuschätzen. Das in RABBIT beobachtete Risiko sollte Behandelnde nicht davon abhalten, Patient:innen, die eine JAKi-Therapie benötigen, mit dieser zu behandeln. Die aufgezeigte Risikoerhöhung sollte sorgfältig gegen das bekannte Malignitätsrisiko bei einer unzureichenden Krankheitskontrolle abgewogen werden. Weder die vorliegenden Ergebnisse aus dem RABBIT-Register noch jene der ORAL Surveillance-Studie können beantworten, ob ein Patient, der mit einem JAKi eine bessere Kontrolle seiner RA-Krankheitsaktivität erreicht als mit anderen Therapieoptionen, mit dieser Behandlung ein höheres Malignitätsrisiko hat als ohne sie. Ärzt:innen sollten daher gemeinsam mit ihren Patient:innen sorgfältig abwägen, ob das beschriebene Malignitätsrisiko die potenziellen Risiken des Absetzens oder des Vorenthaltens einer hochwirksamen Therapie wie JAKi überwiegt. Ein Ausweg aus diesem Dilemma könnte sein, bei JAKi-Therapie von Risikopatient:innen auf eine besonders engmaschige Krebsvorsorge zu achten.

## Fazit für die Praxis

- In einer umfassenden Analyse von Daten aus der Routineversorgung wurde ein insgesamt leicht erhöhtes Risiko für Malignome bei Patient:innen mit rheumatoider Arthritis (RA), die jemals mit Januskinaseinhibitoren (JAKis) behandelt wurden, im Vergleich zu Patient:innen, die jemals mit biologischen krankheitsmodifizierenden Antirheumatika (bDMARDs) behandelt wurden, festgestellt, wobei die Risikoschätzungen mit denen der randomisierten ORAL Surveillance-Studie vergleichbar sind.
- Besonders von der beobachteten Risikoerhöhung betroffen sind Patient:innen im Alter ab 60 Jahren, Patient:innen, die bereits 3 und mehr Behandlungen mit konventionellen synthetischen Antirheumatika (csDMARDs) bekommen haben, sowie Patient:innen mit einer besonders hohen Krankheitsaktivität. Weitere überdurchschnittlich betroffene Gruppen sind Raucher:innen, Männer und Patient:innen mit über 10 Jahren RA-Krankheitsdauer.
- Ärzt:innen sollten im Blick haben, welche ihrer Patient:innen zu einer Gruppe gehören, die mit erhöhtem Malignitätsrisiko bei JAKi-Therapie im Vergleich zu bDMARDs assoziiert ist. Bei diesen Patient:innen sollten sie das Malignitätsrisiko durch die Therapie sorgfältig gegen das Malignitätsrisiko durch eine unkontrollierte Krankheitsaktivität abwägen sowie auf eine individuell angepasste Intensität der Krebsvorsorge achten.
